# Could LASCA-imaging of GB-speckles be applied for a high discrimination and typing of pathogenic bacteria?

**DOI:** 10.1371/journal.pone.0245657

**Published:** 2021-01-28

**Authors:** Onega Ulianova, Sergey Ulyanov, Sergey Zaytsev, Yuriy Saltykov, Alexander Ulyanov, Valentina Feodorova

**Affiliations:** 1 Department of Medical Physics, Saratov State University, Saratov, Russia; 2 Federal Research Center for Virology and Microbiology, Branch in Saratov, Saratov, Russia; Portland State University, UNITED STATES

## Abstract

In this article, the method of analysis of GB-speckles (gene-based speckles) has been adapted to the problem of detecting the differences in a group of genes (usually 5–7), used in Multi Locus Sequence Typing (MLST). This method is based on *s-LASCA* imaging (*s*patial *La*ser *S*peckle *C*ontrast *A*nalysis) of virtual GB-speckle and on the technique of RGB coordinates for GB-speckles, processed by the *s-LASCA* method. A very high sensitivity and accuracy of the new method for detecting gene polymorphism as a great alternative to classical MLST has been demonstrated. The analysis of GB-speckles, obtained for the concatenated sequences of seven genes (*gatA*, *gidA*, *enoA*, *fumC*, *hemN*, *hflX*, *oppA*) of three different *Chlamydia trachomatis* strains (E/Bour, ST94; G/9301, ST95; G/11222, ST94) has been applied as the model. The high efficiency of usage of s-*LASCA*-imaging of GB-speckles has been shown. The data obtained represent a significant progress in digital biology as a whole and improvements in the bio-digitalization of bacterial DNA.

## Introduction

When laser radiation diffracts on random objects (such as rough surfaces or bulk media containing random contamination), the structure of scattered radiation becomes spotty. Randomly placed light spots appear everywhere, varying in size, phase, and intensity of light. It is said that laser speckles are formed [1]. When coherent radiation is scattered by random biological objects, speckles are also formed. Such speckles have been termed biospeckles. This term was introduced for the first time in the article [2]. Usually, biospeckles are used in the analysis of velocity of blood microcirculation [3]. Recently it has been demonstrated [4–8] that the nucleotide sequence also can be transformed into a 2D speckle pattern. Such a principally new kind of speckles is called GB-speckles [5, 7, 8], which is the acronym for gene-based speckles. These GB-speckles can be considered a virtual representation of a nucleotide sequence.

The minimal changes, occurring in the nucleotide sequence, which are caused by natural mutations, produce noticeable changes in the structure of the GB-speckle pattern throughout the whole image area. This makes the detection of a single SNPs (Single Nucleotide Polymorphism) using virtual GB-speckles extra sensitive, while the accuracy of the diagnosis can be increased unlimitedly by increasing the area used in the Fourier transform [9].

Considerable progress in the field of GB-speckles has been achieved in recent years. As it has been shown earlier [4–8, 10], the application of speckle-interferometry and speckle-correlometry to processing GB-speckles will lead both to the significant improvement in the existing bioinformatics tools and to the creation of novel ones. This is critical for improving the methods of laboratory diagnostics of the infectious and non-infectious diseases of humans and animals. The usage of GB-speckles can be viewed as the next step on the way to the era of digital biology [11, 12]. By now, the reference speckle pattern of *omp1* gene of typical wild strains of *Chlamydia trachomatis* of genovars D, E, F, G, J and K and *Chlamydia psittaci* as well has been generated [4, 5]. The statistics of GB-speckles has been particularly investigated by Ulyanov S.S. et al. [5–7]. As it has been shown in the reports [4–8], the usage of such methods of speckle-optics as speckle-correlometry, speckle-interferometry and subtraction of speckle-images allows defining the presence of natural mutations when comparing strains even in the case of a single SNP. It has been demonstrated by Ulyanov S.S. et al. [4] that the appearance of any type of mutations leads to the formation of the system of interferential fringes in the interference pattern when speckle-interferometric technique is being used. This may serve as the basis for the operation of optical processor of genetic information.

The optimization of the algorithm of encoding a nucleotide sequence of bacteria *C*. *trachomatis* into a 2D GB-speckle pattern has been carried out in the reports [4, 6]; it has been shown that the algorithm used in Ulyanov S.S. et al. [5] is close to the optimal one. The method of virtual phase-shifting speckle-interferometry (4-bucket technique) has been efficiently applied [10] to the investigations of polymorphism of two variants of *omp1* gene of *C*. *trachomatis* (namely, strains E/Bour (E1 subtype) and E/IU-4 2 0755u4 (E2 subtype)).

This approach has already been successfully used for the detection of the *C*. *trachomatis omp1* gene of the 11 known subtypes of this bacteria with genetic mutations in the form of either a single SNP or a combination of several SNPs, as previously reported by us—Feodorova V.A. et al. [6].

The nucleotide sequences of genes encoding the production of serine proteases, the *Omptin* family proteins of *Enterobacteriaceae*, which are known to be the causative agents of such infections as salmonellosis, yersiniosis, shigelosis and escherichiosis, have been successfully transformed into the format of GB-speckles. Such genes as pla (*Yersinia pestis*), pgtE (*Salmonella enterica*), sopA (*Shigella flexneri*), ompT and ompP (*Escherichia coli*), have recently been studied using the relevant GB-speckles [7].

A principially new approach in modern bioinformatics has been suggested by Ulianova O.V. et al. [13]: the application of *s-LASCA* technique (spatial Laser Speckle Contrast Analysis) to processing GB-speckles. As it has been demonstrated in the above-mentioned work [13], the using of *s-LASCA* imaging in the processing of GB-speckles allows further increasing the sensitivity of the proposed method in comparison with the classical methods of bioinformatics [14].

Such method of *LASCA* [15] was proposed more than 20 years ago. Previously, this method was used to diagnose the blood flow in normal state and under some pathological changes [16, 17], to monitor of malignant tumor growth, to test the toxicity of the new-generation vaccine against very dangerous diseases [18], and to study the hydrodynamics of microflow in isolated blood vessel in the mesentery of white rats [19]. Relatively recently, essential progress has been achieved in the implementation of *LASCA* method to monitoring bacterial colony growth [20].

MultiLocus Sequence Typing (MLST) is a widespread DNA sequence-based molecular typing method, in which nucleotide sequences of multiple (usually 5–7) housekeeping genes or loci within the bacterial genome should be analysed [21]. MLST is highly discriminatory with the microbial strains, including pandemic variants. Therefore, MLST is successfully used as an important tool for understanding the molecular evolution of microorganisms and is avaiable for global molecular epidemiology worldwide. In fact, MLST provides surveillance and management of disease outbreaks, which is of paramount importance for quickly typing and tracking infectious diseases [21]. It is important to note that the allelic variation at each target locus has been catalogued and standardized for the majority of organisms with a MLST database accessible online at http://www.mlst.net or http://www.pubmlst.org. Therefore, the nucleotide sequences could theoretically be bio-digitalized. On the other hand, a sequence type (ST) or lineage is assigned by comparing the set of alleles to other isolated profiles in the database. This may lead to serious mistakes in handling and difficulties in data analysis interpretation. This article shows that the use of *s-LASCA* imaging method in combination with the generation of GB-speckles is very useful and productive in the analysis of the nucleotide sequences of multiple genes of C trachomatis. The nucleotide sequences for the concatenated sequences of seven genes (*gatA*, *gidA*, *enoA*, *fumC*, *hemN*, *hflX*, *oppA*) used in traditional MLST were converted to GB-speckles and then compared among themselves for three different strains of *C*. *trachomatis* (E/bour, ST94, G/9301, ST 95, G/11222, ST94) those were applied as the appropriate bacterial model.

The purpose of this work is to test whether approaches based on *s-LASCA* processing of GB-speckles work in relation to the analysis of target genes traditionally used in MLST.

## Materials and methods

To generate GB-speckles, the initial sequence of letters (taken from initial nucleotide sequence) is transformed into a sequence of numbers. This process is described in detail by Ulyanov S.S. et al. [4]. The optimisation of transformation algorithms has been done by Feodorova V.A. et al. [6]. Briefly, the process of forming GB-speckles is as follows. Nucleotide sequence is re-coded into a sequence of triads and discrete value *h* is assigned to each possible triad. Then square matrix *H*_*n*,*m*_ is packed from the obtained sequence of *h* values. The physical meaning of the formed matrix *H*_*n*,*m*_ is the local height of virtual scattering surface, reflecting the local content of gene structure. 2D speckle pattern (i.e. GB-speckles), corresponding to the initial nucleotide sequence, is generated using the virtual diffraction of coherent beam with a square profile on the (virtual) rough surface with profile *H*_*n*,*m*_. Two-dimensional discrete fast Fourier transform is applied to compute GB-speckles (Matlab R15b has been used for computing). The precision of GB-speckles computation depends on the size of matrix *H*_*n*,*m*_, which in this case has the dimention of 2048 x 2048 elements.

*S-LASCA* technique has been applied to the processing of GB-speckles. The *s-LASCA* method is based on the analysis of an individual realization of static speckles [3]. As it has already been mentioned, in this case the entire realization of the speckle field is divided into a large number of small square areas, each including, as a rule, 5x5 or 7x7 pixels. For each of the selected small areas the local value of static speckle contrast is calculated, after which the *LASCA* image is constructed.

The local contrast of GB-speckles is calculated using the simplest formula:

$$C=\frac{\sigma_{I}}{\langle I\rangle }$$

where *I* is the instantaneous intensity of GB-biospeckles, varying from point to point; σ_*I*_ is the standard deviation of intensity fluctuations. In this paper, the size of subarea (AS) over each local contrast is calculated and varies in the range from 1x1 pixels up to 50x50 pixels.

## Results and discussion

### GB-speckles and their *s-LASCA* images

**Fig 1:**
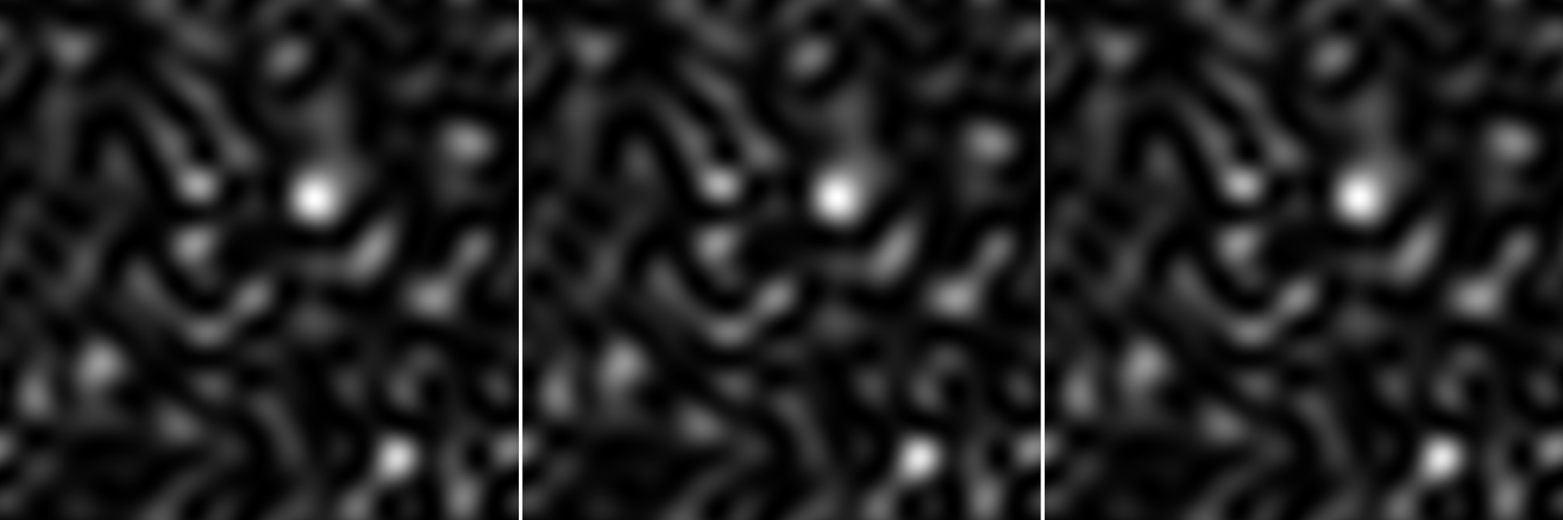
GB-speckles, obtained for *oppA* gene. (a) strain E/Bour, ST94. (b) strain G/9301, ST 95. (c) strain G/11222, ST94.

At first, the GB-speckle patterns have been generated for two individual housekeeping genes of *C*. *trachomatis*, either *gatA* or *oppA*. The dependence of the structure of GB-speckle patterns on the *AS* parameter is shown with the example of *C*. *trachomatis gatA* gene of two different sequence types (STs) (94 and 95, ST94 and ST95, respectively). Raw GB-speckles, obtained for oppA gene, are shown in Fig 1.

**Fig 2:**
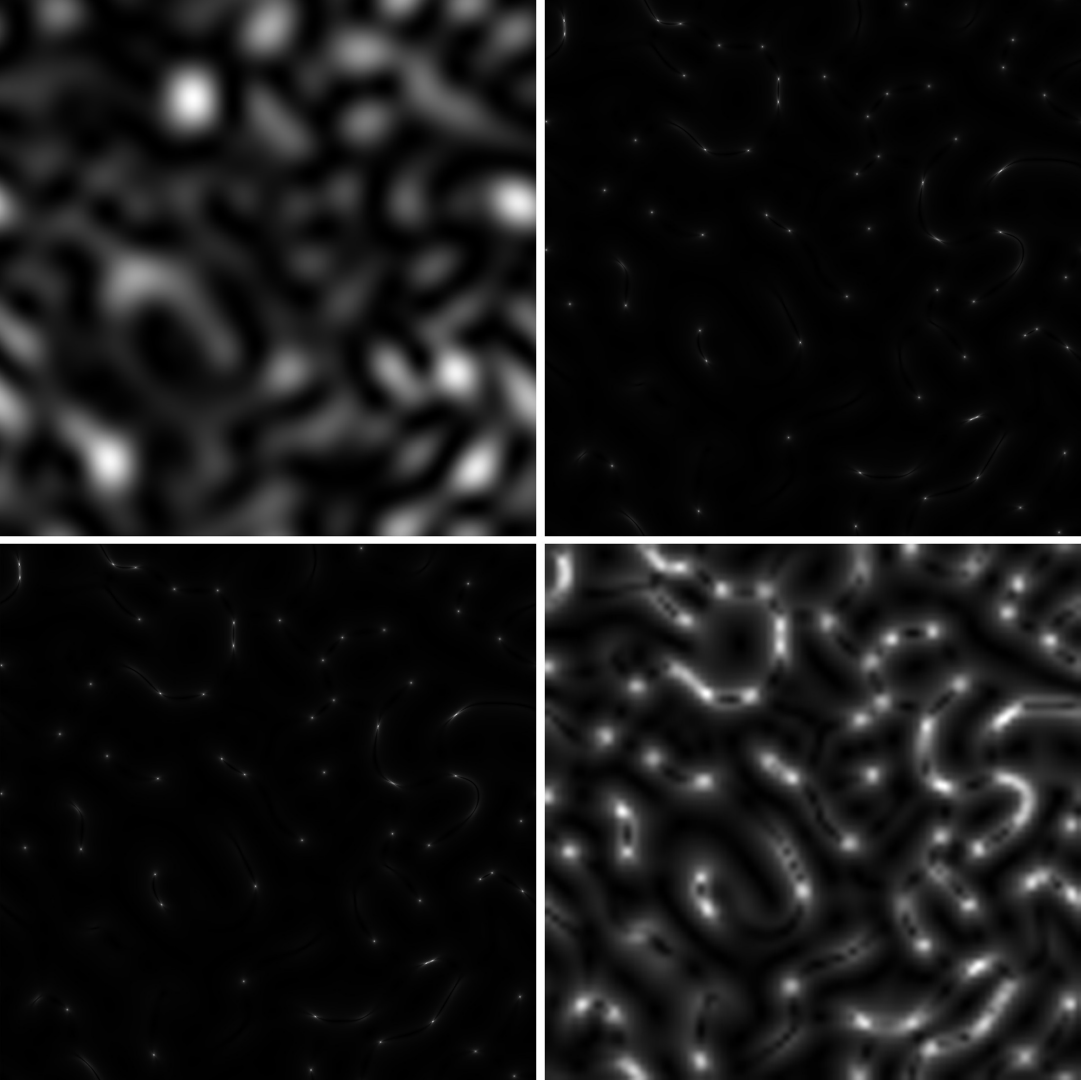
Dependence of the structure of *s-LASCA* images of GB-speckles, obtained for *gatA* gene on the *AS* parameter. (a) *AS* = 1. (b). *AS* = 2 (c). *AS* = 5 (d). *AS* = 50.

Because initial nucleotide sequences for the *C*. *trachomatis oppA* gene of compared strains are identical, the corresponding GB speckles are also completely correlated. This can be seen from Fig 1. It is expedient to remind that the *AS* parameter is the size of subarea over the local spatial contrast is calculating. Fig 2 shows the dependence of the structure of *s-LASCA* images of GB-speckles on parameter *AS*.

GB-speckles and their *s-LASCA* images have been obtained for the nucleotide sequences of the gene *gatA*. A classical picture of statistically inhomogeneous speckle structures [22], formed with a small number of scattering events is observed. In the case of the formation of these speckles their contrast can be greater than unity, because of their spatial statistical inhomogeneity. However, it should be noted that when GB-speckles are formed, their contrast is close to unity, but does not exceed this value. For example, the contrast of speckles shown in Fig 2A equals to 0.964. If the value of *AS* parameter is equal to or greater than 2, the speckles become noticeably elongated. In other words, the speckle structure becomes significantly statistically heterogeneous. In this case, the speckle contrast (see Fig 2B and 2C) increases to a value of 1.54.

This is an essential distinguishing feature of *s-LASCA* images of GB-speckles. The aspect ratio of these speckles on average is about four, see Fig 2B–2D. In other words, the GB-speckles topology changes radically when moving from *AS* equal to one to *AS* equal to two.

Finally, the degree of smoothing of all *s-LASCA* images of GB-speckles increases when the *AS* parameter is also increased. Thus, the speckle contrast drops to the value of 0.928 at *AS* = 5 (Fig 2C) and shows a further decrease to 0.749 at *AS* = 50 (Fig 2D). However, the topology and structural features of all *s-LASCA* images of GB-speckles are preserved in all figures (Fig 2B–2D).

### Dependence of contrast *C* of *s-LASCA* images of GB-speckles on the *AS* parameter for different MLST bacterial profiles

**Fig 3:**
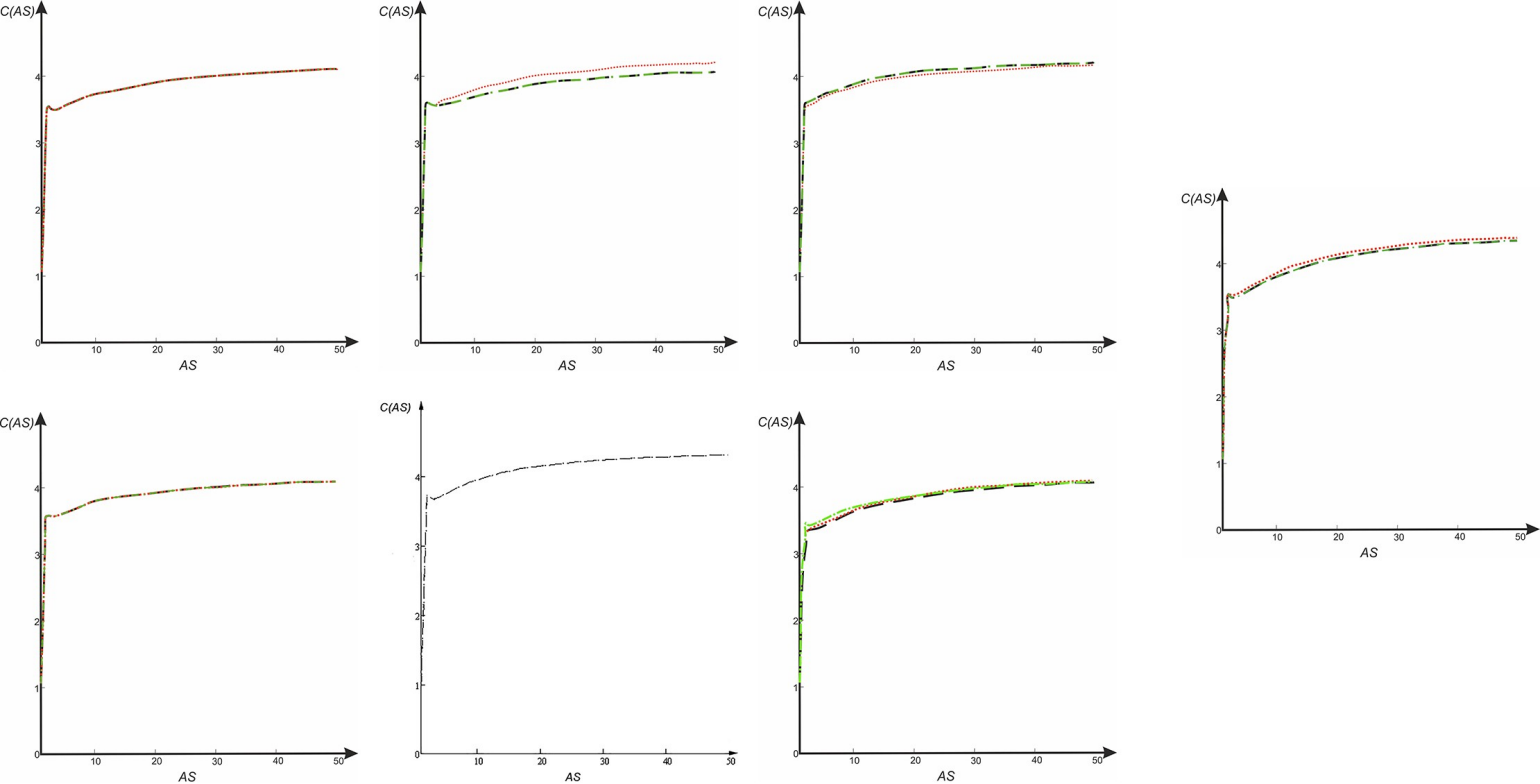
Dependence of contrast *C* of *s-LASCA* images of GB-speckles on parameter *AS*. E/Bour, ST94—dot lines (in red color), G/9301, ST 95 –dashed lines (in black color), G/11222, ST94 –dash-dotted lines (in green color). (a) *gatA*. (b) *gidA*. (c) *enoA*. (d) *fumC*. (e) *hemN*. (f) *hflX*. (g) *oppA*.

The contrast *C* of *s-LASCA* images of GB-speckles, as a function of *AS*, is shown in Fig 3 for seven different housekeeping genes of the similar *C*. *trachomatis* strains (strains E/Bour, ST94, G/9301, ST 95, G/11222, ST94).

It can be seen that all dependencies *C* (*AS*) have an inflection point for the value *AS* = 2, and smoothly tend to the maximum value in the neighborhood of *C* = 4. It should be noted that the maximum value of the spatial contrast *C*_*max*_ is not informative by itself. Evidently, *C*_*max*_ lies within the range [4; 4.22] for all *s-LASCA* images of GB-speckles obtained for all types of genes (*gatA*, *gidA*, *enoA*, *fumC*, *hemN*, *hflX*, *oppA*) and for all compared strains of *C*. *trachomatis* (E/Bour, ST94, G/9301, ST 95, G/11222, ST94).

### Correlation between the *s-LASCA* images of GB-speckles obtained for different *C*. *trachomatis* housekeeping genes

**Fig 4:**
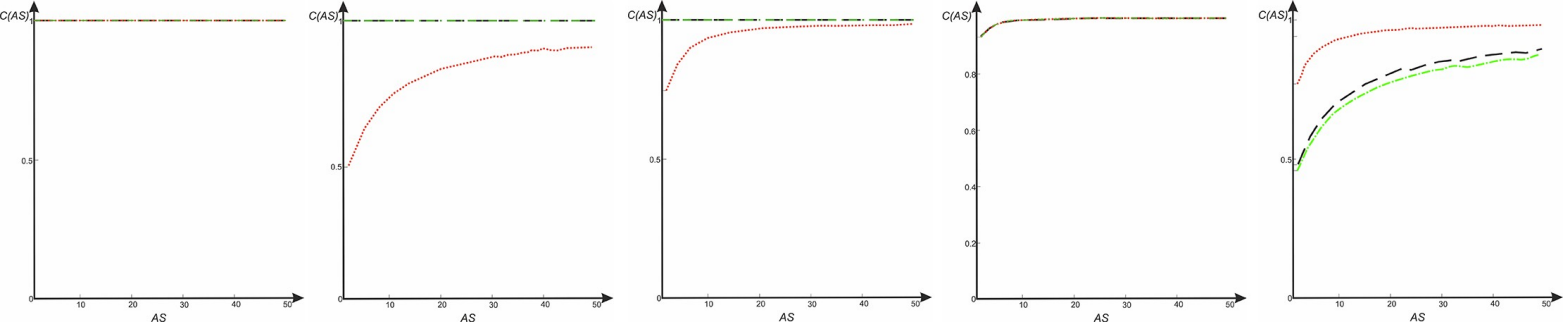
Dependence of the cross-correlation coefficient on the parameter *AS* for s- *LASCA* images of GB-speckles: Red dot lines—correlation between GB-speckles, generated for strain E/Bour, ST94 and for strain G/9301, ST 95, black dashed lines—correlation between GB-speckles, generated for strain E/Bour,ST94 and for strain G/11222,ST94, dash-dotted green lines—correlation between GB-speckles, generated for strain G/9301, ST 95 and for strain G/11222, ST94. (a) Cross-correlation coefficient *CCC* (*AS*) for gene *gatA*, cross-correlation coefficient *CCC* (*AS*) for gene *gidA* and cross-correlation coefficient *CCC* (*AS*) for gene *enoA*. (b) Cross-correlation coefficient *CCC*(*AS*) for gene *fumC*. (c) Cross-correlation coefficient *CCC*(*AS*) for gene *hemN*. (d) Cross-correlation coefficient *CCC* (*AS*) for gene *hflX*. (e) Cross-correlation coefficient *CCC* (*AS*) for gene *oppA*.

The dependence of the cross-correlation coefficient on the *AS* parameter for the s- *LASCA* images of GB-speckles obtained for different target genes for housekeeping genes of different strains of *C*. *trachomatis* is shown in Fig 4.

Fig 4A corresponds to all of these three functions: cross-correlation coefficient *CCC* (*AS*) for gene *gatA*, cross-correlation coefficient *CCC* (*AS*) for gene *gidA* and cross-correlation coefficient *CCC* (*AS*) for gene *enoA*. Clearly, if the compared nucleotide sequences are identical, then the corresponding GB speckles are identical. This situation is observed when comparing *gatA*, *fumC* and *hemN* genes for all three compared strains of *C*. *trachomatis* (E/Bour, ST94, G/9301, ST 95, and G/11222, ST94). In the case where SNP is absent in the compared nucleotide sequences, the correlation coefficient of the corresponding *s-LASCA* images of GB-speckles is equal to unity. However, the appearance of even a single SNP immediately leads to a significant drop in the cross-correlation value. The drop in this value is especially large with the value of the *AS* parameter а equals to 2. In this case, a situation may arise when the correlation coefficient for two different *C*. *trachomatis* strains is equal to unity, whereas the dependence of the correlation coefficient between other strains is complex, but nevertheless similar, see Fig 4B and 4C. Sometimes a specific case may be observed when the *CCC*(*AS*) dependence has a monotonically increasing character and tends to unity in the limit, but is completely identical for all strains, see Fig 4D. Finally, a unique case is possible when the *CCC*(*AS*) function is monotonically increasing and completely identical for each of the compared strains, see Fig 4E.

### A new detection technique using the RGB coordinates method of colored GB-speckles

**Fig 5:**
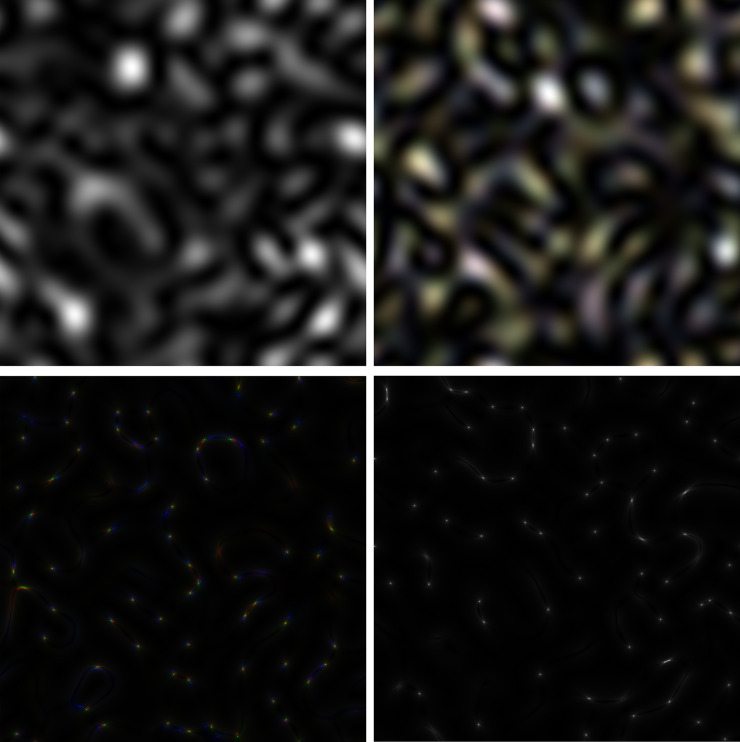
Colored images of GB-speckles. (a) Gene *gatA*. Red component is generated for the *C*. *trachomatis* E/Bour, ST94 strain, green is for the *C*. *trachomatis* G/9301, ST 95 strain, and blue is for the *C*. *trachomatis* G/11222, ST94 strain). Color picture looks like a grey-scale image. (b) Gene *hflX* (Red component is generated for the *C*. *trachomatis* E/Bour, ST94 strain, green is for the *C*. *trachomatis* G/9301, ST 95 strain, and blue is for the *C*. *trachomatis* G/11222, ST94 strain). (c) Colored s-LASCA images of GB-speckles. Gene *hflX* (Red component is generated for the *C*. *trachomatis* E/Bour, ST94 strain, green is for the *C*. *trachomatis* G/9301, ST 95 strain, and blue is for the *C*. *trachomatis* G/11222, ST94 strain). Parameter AS = 2. (d) Colored s-LASCA images of GB-speckles. Gene *gatA* (Red component is generated for the *C*. *trachomatis* E/Bour, ST94 strain, green is for the *C*. *trachomatis* G/9301, ST 95 strain, and blue is for the *C*. *trachomatis* G/11222, ST94 strain). Parameter AS = 2. Color picture again looks like a grey-scale image.

As it is well known, any color image can be represented as a combination of three components: red, green and blue. If all three components are completely identical to each other, then the resulting image will be a gray-scale picture. This very case is depicted in Fig 5A. In this figure, the *LASCA* images of GB-speckles, obtained for the *gatA* gene are presented (red is generated for the *C*. *trachomatis* E/Bour, ST94 strain, green is for the *C*. *trachomatis* G/9301, ST 95 strain, and blue is for the *C*. *trachomatis* G/11222, ST94 strain).

From a formal point of view, the image in Fig 5A is in color. However, it looks grey, since *CCC* is equal to unity for all compared strains. Fig 5B presents images collected from three components for the *hflX* gene, (again, red for the *C*. *trachomatis* E/Bour, ST94 strain, green for the *C*. *trachomatis* G/9301, ST 95 strain, and blue for the *C*. *trachomatis* G/11222, ST94 strain).

These components are very similar to each other, but contain extremely small differences. The appearance of these differences results in the emergence of color spots in Fig 5B. The colorization of images, whose components are based on the compared nucleotide sequences, demonstrates the possibility of using the RGB method of GB-speckles coordinates for application in detecting of polymorphisms of genes, ordinarily utilized in MLST. The appearance of colored fragments is even more noticeable when constructing color images, the components of which are *s-LASCA* images of GB-speckles, see Fig 5C (staining is clearly observed for the *hflX* gene) and 5d (the appearance of staining is absent for the *gat*A gene).

**Fig 6:**
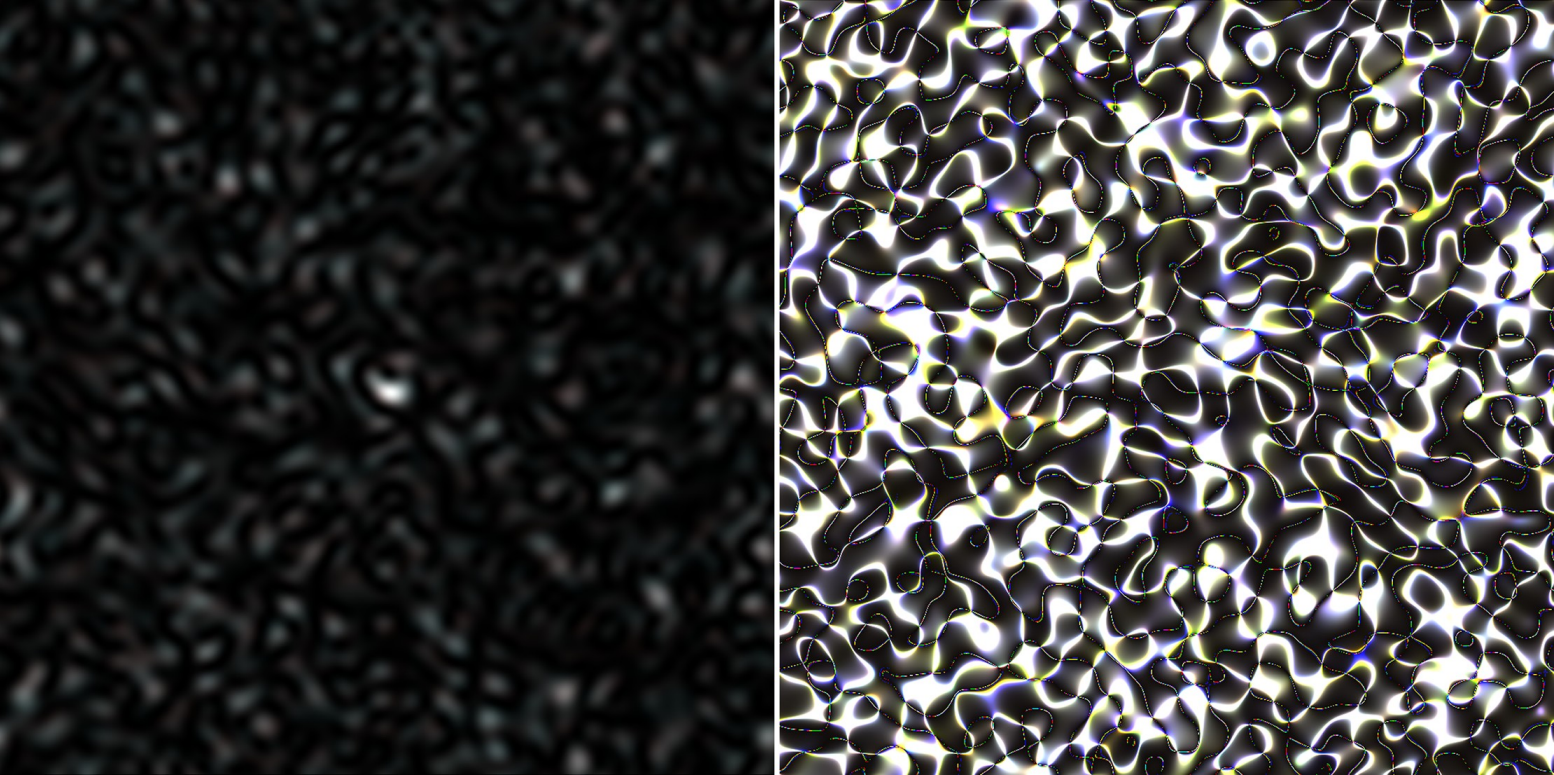
Colored images of GB-speckles, generated for full concatenated nucleotide sequence, combined together from a set of seven genes of house-keepings. (a) Case of raw GB-speckles. (b) Phase map of raw GB-speckles.

Finally, it is expedient to consider the formation of colored speckles, obtained for a full concatenated nucleotide sequence, combined together from a set of seven genes of house-keepings. The GB-speckle-structure for full sequence is shown in Fig 6A. As before, the red component is for the *C*. *trachomatis* E/Bour, ST94 strain, the green component is for the *C*. *trachomatis* G/9301, ST 95 strain, and the blue component is for the *C*. *trachomatis* G/11222, ST94 strain. It is evident that, even GB-speckles built on raw sequence are weakly colored, which indicates the presence of polymorphism in compared sequences. Coloring becomes more evident in the case, when a GB-speckle pattern is formed on the basis of the phase structure of virtual speckles (phase map), see Fig 6B.

## Conclusions

The article is devoted to the study of the correlation properties of GB-speckles, which have been processed by methods of *s-LASCA* imaging. It has been demonstrated that the cross-correlation coefficient between two speckle-structures obtained for two different nucleotide sequences can decrease from unity to 0.75, even in the case of minimal differences between the sequences in only one SNP. Therewith, if there is no polymorphism in the comparing nucleotide sequences, the cross-correlation coefficient between the corresponding *s-LASCA* images of GB-speckles is always equal to unity.

It has been shown that the proposed method has both extremely high sensitivity and high accuracy. It has also been proven that *s-LASCA* images of GB-speckles are a very effective and promising method for analyzing nucleotide sequences, as an alternative to the widely used MLST. Furthermore, it has been demonstrated that the use of combined color in GB-speckles created on a single gene and generated for a total nucleotide sequence as well in the diagnosis of gene polymorphism makes GB-speckles more visible, effective and informative.

This study demonstrates the global progress in digital biology or bio-digitalization of bacterial DNA.
